# Pesticide Exposure and Head and Neck Cancers: A Case-Control Study in an Agricultural Region 

**Published:** 2017-09

**Authors:** Maryam Amizadeh, Mohammad Safari-Kamalabadi, Ghasem Askari-Saryazdi, Marzieh Amizadeh, Hamed Reihani-Kermani

**Affiliations:** 1 *Department of Otorhinolaryngology, Kerman University of Medical Sciences, Kerman, Iran.*; 2 *Plant Protection Research Department, Yazd Agricultural and Natural Resources Research and Education Center, AREEO, Yazd, Iran.*; 3 *Department of Plant Protection, University of Tabriz, Tabriz, Iran.*; 4 *Neurology Research Center, Kerman University of Medical Sciences, Kerman, Iran.*

**Keywords:** Agriculture, Head and neck, Organochlorine, Pesticide, Risk factor

## Abstract

**Introduction:**

Causes of head and neck cancers (HNCs) are multifactorial, and few studies have investigated the association between chemical exposure and HNCs. The objective of this study was to investigate associations between HNCs, agricultural occupations, and pesticide exposure. The potential for the accumulation of pesticides in the adipose tissue of patients was also investigated.

**Materials and Methods:**

A structured questionnaire was used to collect information on demographics, occupation, and exposure to pesticides in a hospital-based case-control study. Pesticide residue in the adipose tissue of the neck in both cases and controls was also monitored via gas chromatography–mass spectroscopy.

**Results:**

Thirty-one HNC cases were included in this study as well as 32 gender-, age-, and smoking-matched controls. An agricultural occupation was associated with HNC (odds ratio [OR], 3.26; 95% confidence interval [CI], 1.13–9.43) after controlling for age, sex, and smoking. Pesticide exposure was associated with total HNC cases (OR, 7.45; 95% CI, 1.78–3.07) and larynx cancer (OR, 9.33; 95% CI, 1.65–52.68). A dose-response pattern was observed for HNC cases (P=0.06) and larynx cancer (P=0.01). In tracing the pesticide residue, five chlorinated pesticides, namely dichlorodiphenyltrichloroethane (DDT), dichlorodipheny-ldichloroethane (DDD), dichlorodiphenyldichloroethylene (DDE), dieldrin, and lindane, were identified in the adipose tissue. Chlorinated pesticide detection was significantly associated with HNC (OR, 3.91; 95% CI 0.9–0.16.9).

**Conclusion:**

HNCs were found to be associated with pesticide exposure after controlling for confounders. A high education level was identified as a modifying factor decreasing the risk of HNCs. Further studies with larger number of subjects are recommended to assess these relationships in greater detail.

## Introduction

Cancer is one of the most important health problems all over the world, and the third highest cause of death in Iran (1). Head and neck cancers (HNCs) are defined as malignant tumors of the airways and upper digestive system (2), and are known to be the sixth most common cancer in the world (3). Cancers of the head and neck are categorized based on the area of the head or neck in which they originate, including the oral cavity, nose, pharynx, larynx, paranasal sinuses and nasal cavity, salivary glands, and thyroid. Larynx cancer is the most widespread type of HNC (4). HNCs accounted for an annual incidence of 690,000 cases (4.9% of all cancers) and almost 375,000 deaths (4.6% of all deaths from cancers) in the world in 2012 (5).

The causes of the different types of cancer are multifactorial and include physical, chemical and biological agents; for example, radiation, solvents, and pathogens (6). Smoking and alcohol are known to be the most common risk factors for HNCs (7). A considerable number of cancer risk factors have been reported in relation to various pesticides (8–10), but few associations between pesticides and cancer have been sufficiently studied in human populations. In particular, very few studies have examined the relationship between pesticides and HNCs, although some researchers have found a positive correlation between pesticides and HNCs (11,12). Pesticides are used for killing pest organisms and include substances that kill weeds (herbicides), insects (insecticides), fungi (fungicides), and rodents (rodenticides), for example. Pesticides have been linked to a wide range of human health hazards, ranging from short-term impacts such as headaches and nausea to chronic impacts such as cancer, allergies, neurological disorders, reproductive disorders, and endocrine disruption (13-15). Pesticides are mostly used in agriculture areas and may affect people who are involved with agricultural practice.

**Fig1:**
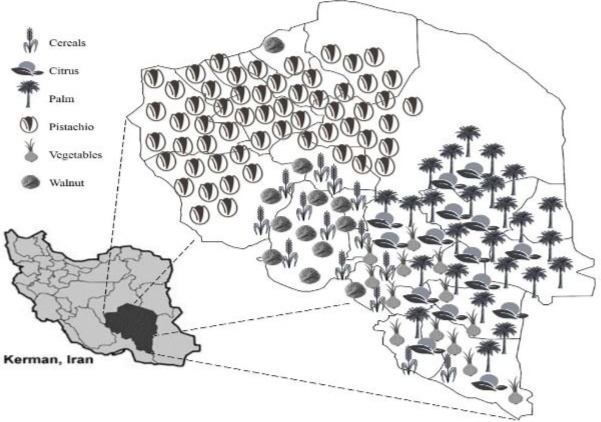
Schematic representation of crop distribution in the Kerman province (Iran

Kerman is the largest province of Iran, and is located in the south-east of the country. Over last 4 decades, the province has enjoyed an economy based primarily on agricultural activities. Kerman is one of the main agricultural regions of Iran and is the largest pistachio producer area all over the world (*ca.* 213300 ha). It is also one of the main regions producing citrus (*ca.* 42000 ha), palm (*ca.* 159300 ha), and vegetables (*ca.* 88300 ha) in Iran. Other fruit trees such as walnut and also cereals are among the other important products from the province (16). A distribution map of the various crops grown in the province of Kerman is shown in Figure 1. Due to the wide variety of crop products and large cultivation area in the Kerman province, various types of pesticides are used in this area (17).

Most studies investigating the role of pesticides and cancer risk have been focused on cancers other than HNCs. Data on the relationship between exposure to pesticide and HNCs are rare. Moreover, the actual status of any association between pesticide exposure and disease in Iran is not well documented. The objective of the current study was to determine the relationship between exposure to pesticide and HNCs in the province of Kerman, with it’s a substantial agricultural labor force. In addition, the study also evaluated the possibility of pesticide accumulation in the fat tissue of cancer patients and controls.

## Materials and Methods

A case-control study of HNCs was designed and conducted in Shafa Hospital of Kerman (Kerman, Iran). The current study was conducted from May 2011 to March 2015.

*Cases and controls*

The case group consisted of HNC patients who were candidates for neck surgery for various reasons, or HNC patients in whom sampling of the neck adipose tissue was possible. The control group were selected on the basis of benign lesions in the head and neck area. Since smoking and alcohol are known risk factors for HNC, in order to eliminate the effects of this factor all individuals in both the case and control groups were smokers and non-alcoholic.

*Questionnaire*

In order to collect data from both cases and controls, a structured questionnaire was developed based on a study by Kokouva et al. (18) (Appendix 1). The questionnaire included four different sections: 1) demographics,2) residence,3) occupation, 4) exposure and agricultural practice. The demographic section included age, sex, and level of education. The residence section included full address and residential level (urban and suburban or rural). In the occupational section, detailed information on the history of agricultural activities was reported. Individuals who reported at least 1 year of agricultural occupation were asked to complete the pesticide exposure section. Information collected included type of crop, pest and agricultural activities (farm manager, worker, or rancher) duration of farming, and the area of farming surface. The subjects were asked to provide the name of pesticide products, number of applications per year, method of application, and use of personal protective equipment (PPE). Additional information such as accuracy of the pesticide label, eating and smoking habits during pesticide application, and showering after spraying was also collected. The interviews were conducted by a trained ear, nose, and throat (ENT) resident.

Due to changes in pesticide exposure over time, an expert (pesticide toxicologist) evaluated each questionnaire and data collected regarding pesticide exposure and handling practice as well as commercial names of pesticides and method of application. Chemicals were grouped according to the target pest into three major classes: insecticides, fungicides, and herbicides. The level of pesticide exposure was based on protocols presented by Kokouva et al. (2011), with some modification (17). 

Residential, occupational, and agricultural practice information was used for exposure categorization. Participants who had never worked within agricultural sectors as their main or secondary occupation and had lived in urban and suburban regions were classified in the “No/Low” exposure category. The following equations were used for determining the “Medium” and “High” exposure categories: number of treatments per year × total years of pesticide application × cultivation area (ha).

 A value of 200 (application/ha) was set as the cutoff point between “Medium” and “High” exposure levels. Some modification factors were applied before the final categorization of “Medium” and “High” exposure groups. These factors were application of pesticides by the person (×1.3), using reduced exposure equipment during application (× 0.8), and use of PPE (×0.9).

*Fat tissue biopsy*

Adipose tissue biopsy was performed through a cervical incision. A segment of adipose tissue (ca. 2×1.5×1.5 cm) was resected. The adipose tissue was placed in a micro-tube and immediately frozen in liquid nitrogen. The samples were kept at −20°C until analysis.

*Pesticide extraction*

Pesticide residues from the adipose tissue were extracted according to the methods of Waliszewski et al. and Covaci et al. , with some modifications (19, 20). Approximately 1g of each sample was mixed with 0.5 g of anhydrous sodium sulfate, then the mixture was homogenized and extracted with 15 ml of hexane and diethyl ether (1:1) in a test tube with a glass stopper (2.5-cm diameter and 25-cm height) and stirred for 15 min. The solvents were then transferred to a clean tube. The extraction was continued by adding the solvent three times. The extracted mixture was concentrated by removal of the solvents using a vacuum pump. Then, the extract was mixed with 1 ml of concentrated sulfuric acid. The contents were then shaken for 30 s and the tube allowed to stand still for 5 min for phase separation. The supernatant was then dried by passing it through a 2-cm layer of sodium sulfate, which was then washed with diethyl ether. The final extract was concentrated to a small volume (ca 1 ml) and transferred to a darkened tube. Finally, 1 μl of the extract was injected into gas chromatograph for qualitative analysis.

*Gas* *chromatography–mass* *spectrometry analysis: *For gas chromatography–mass spectrometry (GC–MS) analysis, an Agilent 6890 gas chromatograph with a 30 m–0.25 mm HP-5MS capillary column coupled with an Agilent 5973 mass spectrometer (Agilent Technologies, Palo Alto, CA) was used. The temperature program was 50°C (5 min) to 270°C at 7°C/min, held for 5 min in 270°C; the carrier gas was helium. Final detection of the components was based on a comparison between their mass spectra and those of the internal Wiley GC–MS spectral library.

*Statistical analysis*

Standard statistical procedures were carried out using Stata version 12 (StataCorp, College Station, TX, USA). Descriptive analysis was conducted for variables. The chi-square test was used to analyze qualitative data. Logistic regression was used to control for confounders and to identify risk factors.

*Ethics* The study was approved by the ethics committee of Kerman University of Medical Sciences. The participants also made a verbal agreement, and non-monetary incentives for participation were offered. Information collected through the questionnaires was also kept confidential.

## Results

Overall, 31 cases and 32 controls were included in this study. Characteristics of the study population, including sex, age, agricultural occupation, pesticide exposure, and education level, are presented in Table 1. There were no significant differences between cases and control in terms of gender, age structure, or rate of smoking (Table.2). Due to the unequal distribution of these factors in the occurrence of cancer amongst cases and controls, the factors were considered confounding factors. Therefore, further studies were not performed based on these factors.

**Table 1:** Description of the study population

**Characteristics**	**Cases**		**Controls**		**P** **-value**
Sex					
Male	30	96.8%	28	87.5%	
Female	1	3.2%	4	12.5%	0.173
Age (years)					
21–40	3	9.7%	7	21.9%	
41–60	21	67.7%	17	53.1%	
61–80	7	22.6%	8	25%	
Age (years)†	54.5 (±9.08)		53.9 (±13.26)		0.84
Smoking (PY)†*	28.2 (±24.49)		23.3 (±18.83)		0.37
Agriculture occupation					
Yes	23	74.2%	14	43.7%	
No	8	25.8%	18	56.3%	
Pesticide exposure					
No	6	19.3%	17	53.1%	
Low	6	19.3%	10	31.3%	
High	19	61.3%	5	15.6%	
Education					
Illiterate	15	48.4%	5	15.6%	
<High school	13	41.9%	14	43.8%	
≥High school	3	9.7%	13	40.6%	
Total	31		32		

† means (±SE)

* PY: pack-year = (the number of cigarettes smoked per day / 20) × years of smoked

**Table 2:** Characteristics of the case and control groups

**Characteristic**		**Case**	**Control**	**P** **-value**
Sex	Female	1	4	
	Male	30	28	0.173
Age (years)†		54.5 (±9.08)	53.9 (±13.26)	0.84
Smoking (PY)†*		28.2 (±24.49)	23.3 (±18.83)	0.37

† means (±SE)

* PY: pack-year = (the number of cigarettes smoked per day / 20) × years of smoked

The educational status of the case and control groups is summarized in Table 3. The level of education in the control group was significantly higher than that in the case group. Further, as shown in Table 3, statistical analysis revealed a significant association between exposure to pesticide and HNC cases, with an odds ratio (OR) of 1.47 and 7.45 for low- and high-exposure risks, respectively. A dose-response effect was observed for risk of HNC and was statistically significant for cases with a high-exposure risk (P=0.006). The results for laryngeal cancer, as the most common among HNCs, were similar (Table.4).

**Table 3:** Agricultural occupation, exposure risk, and educational level of HNCs compared with heathy controls

**Characteristic**	**Case**	**control**	**OR (95 % CL)**	**P value**
Agricultural occupation				
No	8	18		
yes	23	14	3.26 (1.13 – 9.43)	0.03
Exposure level				
No exposure	6	17		
Low exposure	6	10	1.47 (0.034 - 6.32)	0.6
High exposure	19	5	7.45 (1.78 – 31.07)	0.006
Education level				
< high school	15	5		
≥ high school	16	27	0.19 (0.06-0.64)	0.007

**Table 4:** Analysis of pesticide exposure risk among Larynx cancer cases and controls

**Exposure level**	**Case**	**Control**	**OR (95% Cl)**	**P value**
No exposure	4 (19.05 %)	17 (53.1 %)		
Low exposure	3 (14.29 %)	10 (31.3 %)	1.36 (0.14 – 5.22)	0.87
High exposure	14 (66.67 %)	5 (15.6 %)	9.33 (1.65 – 52.68)	0.01
Total	21	32		

Based on GC–MS analyses, only five chlorinated insecticides were detected in the analyzed samples, with none of the other pesticides detected. The number of detected cases in cancer patients was significantly greater than in the control group (Table.5). In the case group, organochlorine pesticides (OCPs) were detected in 11 of the subjects, compared with only three in the control group.

A summary of results for OCP detection is presented in Table 6. Only five OCPs, namely dichlorodiphenyltrichloroethane (DDT), dichlo- rodiphenyldichloroethane (DDD), dichlorodiph enyldichloroethylene (DDE) (two metabolites of DDT), dieldrin, and lindane, were detected. The number of cases of pesticide detected differed significantly between the groups in terms of DDT, DDE, and dieldrin pesticides (P<0.05).

**Table 5:** Chlorinated pesticide detection distribution in case and control groups.

**GC–MS result**	**Case**	**Control**	**OR (95% CI)**	**P-value**
Non-detected	20 (64.50%)	29 (90.50%)		
Detected	11 (35.50%)	3 (9.5%)	3.91 (0.90–16.90)	0.06
Total	31	32		

**Table 6:** Analysis of chlorinated pesticide detection in adipose tissue of HNC cases and controls

**Pesticide**	**GC–MS result**	**Case**	**Control**	**Total**	**P** **-value**
DDT	Non-detected	26	31	57	
	Detected	5	1	6	0.07
DDE	Non-detected	28	32	60	
	Detected	3	0	3	0.07
Dieldrin	Non-detected	28	32	60	
	Detected	3	0	3	0.07
Lindane	Non-detected	27	31	60	
	Detected	4	1	5	0.15
DDD	Non-detected	26	30	60	
	Detected	5	2	7	0.21

## Discussion

This study found significant associations between agricultural occupation and risk of HNCs. It seems that increasing exposure to agrochemicals is the most important risk factor for cancers among agricultural occupations. As we found in this study, there were also significant associations between exposure to pesticides and HNCs after controlling for confounding factors. A dose-response relationship was also seen in all HNC cases, especially larynx cancer cases. Many chemicals such as pesticides are thought to have carcinogenic potential in animals and humans (8,10). A variety of chemical pesticides are used in the main groups of crops in Kerman. The general pattern of pesticide application in the province is briefly summarized in Table 7. These data are taken from the interview with farmers and recommended protocols of the Plant Protection Organization of Iran (17). The adverse effects of some of these pesticides have been studied in the literature. Some of the widely used pesticides in Kerman, such as amitraz, dichlorvos, diazinon, permethrin, chlorothalonil, meta-sodium, 2,4-D-glyphosate (2,4D), and 2-methyl-4-chlorophenoxyacetic acid (MCPA), were classified as probable/ possible human carcinogens by the United States (US) Environmental Protection Agency (EPA) and/or *International Agency for Research on Cancer* (IARC; Table.7) (21,22). Organophosphates (OPs) are the main group of pesticides used in Kerman, and many OPs and their metabolites have been shown to be carcinogenic (23–26). Cabello et al. demonstrated that OPs can cause mammary tissue carcinogenesis in rats (27), while Isoda et al. reported an increased breast cancer risk as a consequence of exposure to dichlorvos, an OPs compound (28). Another widely used OP compound in Kerman is fenitrothion, which is thought to increase the androgen receptor activity of human cells in vitro (29). Several diseases such as prostate and breast cancer are also associated with alterations in androgen receptor functions (30,31). Chlorpyrifos is also suggested as a potential genotoxin on the human lymphocyte cell (32). Amer and Aly have investigated the genotoxicity of 2,4-D, a commonly used herbicide, on certain mice cells (33), and suggested 2,4-D as a potential genotoxic *in vivo*. Amitraz is a formamidine insecticide/acaricide widely used in pistachio orchards over the past 2 decades, although it has been banned in recent years (17). Amitraz is a highly liposoluble compound that is quickly absorbed thought the skin and mucous membrane, and is consequently potentially hazardous for human and animals. Genotoxicity of this pesticide has also been reported (34,35). Genotoxic potentiality is a primary risk factor for long-term effects such as carcinogenicity and reproductive toxicity (36,37).

**Table 7:** Pattern of pesticides application in Kerman province and the carcinogenicity classification of these agrochemicals

	** Carcinogenesis classification**					
**Pesticides**	**Register in Iran**	**Chemical class**	**Usage amount in Kerman (kg ai/ year)**	**Main target crops** *	**EPA** †	**IARC** ‡
**Insecticides ** **and Acaricides**						
Abamectin	1998	Avermectins	10000	Ct, Vg	NL	NL
Amitraz	1999-2012	Formamidines	140000	Ps	SECP	NL
Bromopropylate	1970	Benizlates	20000	Ct, Pl, Vg	NL	NL
Chlorpyrifos	1976	Organophosphates	120000	Ps, Ct, Vg	E	NL
Diazinon	1968	Organophosphates	30000	Ps, Ct, Vg,	E	2A
Dichlorvos	1968	Organophosphates	40000	Vg	SECP	2B
Endosulfan	1968-2008	Organochlorine	150000	Ps	E	NL
Ethion	1968	Organophosphates	150000	Ps	E	NL
Fenitrothion	1968	Organophosphates	120000	Ps, Cr, Vg	E	NL
Heptenophos	1979	Organophosphates	10000	Vg	NL	NL
Imidacloprid	1998	Neonicotinoids	14000	Ps, Vg	E	NL
Oxydemeton methyl	1968	Organophosphates	15000	Vg, Ps	E	NL
Permethrin	1978	Pyrethroids	100000	Ps	B2	3
Phosalone	1968	Organophosphates	120000	Ps	E	NL
**Fungicide**						
Carbendazim	1975	Benzimidazole	15000	Vg, Cr	C	NL
Carboxin	1974	Carboxamide	10000	Cr	E	NL
Chlorothalonil	1994	Chloronitrile	25000	Vg	B2	2B
Mancozeb	1970	Dithiocarbamates	60000	Vg	B2	NL
Metalaxyl	1992	Acylalanine	8000	Vg	E	NL
Metam sodium	1968	Dithiocarbamates	20000	Vg	B2	NL
Thiram	1969	Dithiocarbamates	15000	Vg, Cr	E	3
**Herbicide**						
2,4-D	1968	Phenoxycarboxylic	4500	Cr	D	2B
Glyphosate	1977	Glycine derivative	200000	Ps, Ct, Pl	E	2A
MCPA	1968	Phenoxycarboxylic	3500	Cr	E	2B
Paraquat	1968	Bipyridylium	50000	Ps, Ct, Pl	E	NL

* Ps=Pistachio; Ct=Citrus; Pl=Palm; Vg=vegetable; Cr=Cereals

† B2=Likely to be carcinogenic to humans, C=Possible human carcinogen, SECP=Suggestive evidence of carcinogenicity, but not sufficient to assess human carcinogenic potential. D=Not classifiable as to human, E=Evidence of non-carcinogenicity for human

‡ 2A=probably carcinogenic to humans, 2B=possibly carcinogenic to humans, 3=Not classifiable as to its carcinogenicity to humans

Interestingly, it should be noted that the use of detected OCPs has been restricted in Iran for the past 20 years or so. Nonetheless, these pesticides were wildly used for pest control in the agricultural, livestock, and health sectors between 1960 and 1990 in Iran. Pesticides have predominantly been used for controlling mosquitoes in large areas such as urban and suburban areas, and villages (38,39). Nearly all of the OCPs have been phased out of use in Iran and many other countries because their long residual life causes environmental hazards (30). These compounds are very stable in the environment and can remain in soil and water for a large number of years and enter the food chain (39,40).

Biomagnification factors for OCPs are reported to be up to 70,000 times (15). Thus, a huge amount of these compound can enter human food sources and ultimately be traced in the human body, without any direct contact with these toxins. Bioaccumulation and bioconcentration of these compounds in the human adipose tissue has also been reported in the literature (12,19,20,41). The detected OCPs are also listed as carcinogenic agents by the EPA and IARC (21,22). *In vitro* studies have shown OCPs to promote the growth of breast, ovarian and prostate cancer tumor cells via alteration of tumor suppressor genes (42–44). Some case-control studies investigated the relationship between burden of OCPs in a woman’s body and breast cancer risk. Researchers have found a positive correlation in this issue and have reported that women with higher burdens were more likely to develop these cancers (45,46).

Pesticides such as 2,4-D, chlorpyrifos, glyphosate, diazinon, endosulfan, fenitrothion, lindane, dieldrin, DDT, DDE, mancozeb, permethrin and thiram are also defined as endocrine disruptors (ED) (21,26,47). Endocrine disruption refers to a mechanism of toxicity that hampers the hormonal communication of cells, tissues and organs, leading to a wide variety of adverse health consequences such as reproductive abnormalities, reduction in fertility and fecundity, alteration in sex ratios, spontaneous abortion, precocious puberty, polycystic ovary syndrome, impaired immune function, neurobehavioral disorders, and a wide range of cancers (26). Recently, exposure to ED pesticides has been demonstrated in the etiologies of several different cancers (45,48). Chemicals with ED properties can directly affect the DNA and cause changes that may result in abnormalities and cancers in the affected organs (49,50).

Very few epidemiologic studies have been able to evaluate cancer risk for a specific agrochemical. Several studies of field-related exposure have reported increasing risk of cancer such as childhood cancer (51), breast cancer (52), lymphohematopoietic cancer (18), lung cancers, and hormone-related cancers (53). Van Maele-Fabry and Willems found a positive and strong correlation between pesticide exposure and prostate cancer using meta-analysis studies (54), while human epidemiological studies have linked 2,4-D, to endocrine related cancers (55,56). However, no significant association between certain cancers and pesticide exposure was reported in some epidemiological studies (53,57,58). Thus far, strong causal links have not been confirmed between pesticides and human cancers, and most of the estimations are based on epidemiological studies and experiments on animals.

Literacy levels in this study differed between the two groups. A higher level of education increases the awareness of the chemical user, and may make the user more likely to read the chemical labels and use with greater protection. This is consistent with the findings of other researchers referring to the level of education as a potential factor in reducing exposure to different toxins (59,60).

Although all the patients in this study were smokers, and smoking was not significantly different between the groups, some points should be considered. First, smoking in pesticide-contaminated areas (e.g. during spraying) increases the possibility of allowing pesticides to enter the smoker’s body (61,62). Furthermore, pesticides may induce immune alterations and oxidative stress, and may reduce glutathione S-transferase activity in humans and animals. Glutathione conjugation is the main detoxification pathway for benzopyrene epoxides in the lung. This pathway may offer an explanation for the possible co-carcinogenicity of pesticides in combination with polycyclic hydrocarbons from exposure such as smoking (58).

Pesticide exposure is complex because many pesticides are mutagenic, teratogenic, or carcinogenic, whereas others are not. In this respect, it must be noted that extrapolation and generalization of the results is difficult due to the different pesticide formulations used and the complex combinations applied, depending on the region, crop, and season, etc. Formulations are complex and sometimes have confidential proprietary information. Formulated products may contain solvents, emulsifiers, carriers, and dispersants (15), for which their biological activity on humans has not been studied well. In addition, there are many possible methods of exposure, including ingestion, dermal, inhalation and ocular. Different exposure pathways can lead to variable amounts of pesticides in the human body and thus cause different biological effects. Furthermore, mixed or alternating use of pesticides in a season may also give rise to complications. These interactions can synergistically enhance the adverse effects of the chemical. Understanding of these interactions is complicated and not well studied. Information on the particular adverse effects of a defined compound is not enough to adequately evaluate the real genotoxic risk related to complex mixtures. Using higher doses of the chemical or increasing application frequencies in pest outbreak periods may also result in higher pesticide exposures. Using stable agrochemicals such as OCPs also increases human exposure risk through long-term maintenance in the environment as well as entering the food chain (15–39). Some other confounding factors may also obscure links between pesticides and diseases. For example, although farmers are exposed to many pesticides, they also have a lot of physical exercise and sun exposure, two factors known to protect against cancers (63–66). These phenomena may change the results and make interpretation of the results difficult.

A limitation of this study was the small number of subjects, and the inclusion of more people is recommended for future studies. Another limitation was the lack of sufficient non-smokers with HNCs, in whom factors other than smoking could be examined with greater certainty. Another shortcoming of the study was the inability to trace the quality of pesticides in adipose tissue. Monitoring of pesticides residue in the blood serum allows a wider range of pesticides to be traced and is recommended for future studies. Even providing blood samples from farmers in the fields may lead to better results for detecting unstable pesticides. The analysis of blood serum is recommended for future studies. Quantitative analysis of pesticide residue may also allow more definitive results using pesticide standards.

## Conclusion

Previous studies have suggested that the causes of HNCs are multifactorial. The present study revealed an association between pesticide exposure and HNCs, especially larynx cancer, after controlling for confounders. Moreover, a dose-response effect was observed. The results also showed bioaccumulation of OCPs in the human adipose tissue with significant trends in patients suffering from cancer. Further studies are required to confirm whether or not exposure and bioaccumulation of pesticides is a significant determinant in HNCs. It seems the increasing awareness of farmers and pesticide users, particularly the increase in education, the availability of new and effective spraying equipment, and the introduction of PPE and safer pesticides, has decreased exposure risk and reduced related diseases. Finally, further studies using larger samples and different areas are recommended in order to achieve more conclusive results.
